# Supplementary material for: Structural and Functional Responses of the Heteromorphic Leaves of Different Tree Heights on Populus euphratica Oliv. to Different Soil Moisture Conditions
Source: Plants (Basel). 2022 Sep 12;11(18):2376. doi: 10.3390/plants11182376 (PMC9505870; doi:10.3390/plants11182376)
Supplement: Supplementary file 1 [file plants-11-02376-s001.zip › plants-1914658-supplementary.pdf]

Supplementary Table S1. General information of the sample plot (mid-July).

Sample area	Underground water level (m)	Soil water content(%)	Air temperature (℃)	Air humidity (%)
Alar	1.5	21.43	31.31	48.8
Shaya	5.0	4.37	36.65	15.0

Note: Shaya, soil drought stress; Alar, suitable soil moisture conditions.

Supplementary Table S2. Correlation analysis between tree height and structural and functional traits of heteromorphic leaves under suitable soil moisture conditions.

R	H	LI	LA	LT	SLA	PT	PSR	MVBA	MXA	VBA/XA	VA	Pn	Tr	Ci	Gs	RWC	LWP	WUEi	δ^{13} C	Pro	MDA	SS	SP	ABA	GA3	IAA
LI	-0.82**	1																								
LA	0.92**	-0.69**	1																							
LT	0.69**	-0.67**	0.59**	1																						
SLA	-0.69**	0.42*	-0.70**	-0.36	1																					
PT	0.83**	-0.71**	0.71**	0.61**	-0.64**	1																				
PSR	0.41*	-0.35	0.27	0.17	-0.25	0.54**	1																			
MVBA	0.67**	-0.23	0.68**	0.51**	-0.39	0.59**	0.17	1																		
MXA	0.79**	-0.62**	0.73**	0.56**	-0.66**	0.63**	0.32	0.51*	1																	
XA /VBA	0.58**	-0.41*	0.46*	0.43*	-0.24	0.53**	0.12	0.57**	0.15	1																
VA	0.85**	-0.55**	0.69**	0.48*	-0.62**	0.81**	0.38	0.75**	0.57**	0.76**	1															
Pn	0.66**	-0.52**	0.58**	0.35	-0.44*	0.52**	0.36	0.44*	0.58**	0.40*	0.60**	1														
Tr	0.40*	-0.42*	0.49*	0.26	-0.19	0.12	-0.02	0.10	0.25	0.21	0.15	0.58**	1													
Ci	-0.71**	0.60**	-0.55**	-0.59**	0.46*	-0.61**	-0.28	-0.43*	-0.60**	-0.52**	-0.64**	-0.53**	-0.23	1												
Gs	0.74**	-0.74**	0.56**	0.36	-0.52**	0.72**	0.62**	0.26	0.55**	0.40*	0.68**	0.60**	0.19	-0.62**	1											
RWC	-0.52**	0.27	-0.56**	-0.41**	0.52**	-0.49*	-0.44*	-0.45*	-0.37	-0.33	-0.43*	-0.38	-0.34	0.17	-0.28	1										
LWP	-0.35	0.15	-0.31	-0.12	0.27	-0.24	-0.17	-0.33	-0.31	-0.20	-0.41*	-0.38	-0.30	0.03	-0.16	0.07	1									
WUEi	0.04	0.05	0.09	-0.27	-0.16	0.19	0.12	-0.01	0.03	-0.07	0.13	-0.14	-0.41*	-0.01	0.09	0.08	-0.16	1								
δ^{13} C	0.58**	-0.40*	0.62**	0.32	-0.74**	0.48*	0.12	0.32	0.56**	0.26	0.46*	0.08	0.01	-0.38	0.43*	-0.25	-0.03	0.11	1							
Pro	0.62**	-0.58**	0.57**	0.47*	-0.35	0.49*	0.30	0.23	0.31	0.63**	0.47*	0.51**	0.52**	-0.43*	0.54**	-0.40*	-0.25	-0.01	0.31	1						
MDA	0.93**	-0.69**	0.90**	0.63**	-0.71**	0.79**	0.43*	0.67**	0.69**	0.58**	0.82**	0.73**	0.48*	-0.57**	0.65**	-0.60*	-0.44*	0.06	0.52**	0.66**	1					
SS	0.26	-0.33	0.29	0.29	-0.01	-0.03	-0.13	0.05	0.32	0.23	0.04	0.21	0.59**	-0.23	-0.08	-0.02	-0.09	-0.45*	0.14	0.37	0.24	1				
SP	0.41*	-0.27	0.41*	0.56**	-0.14	0.17	0.25	0.52**	0.34	0.23	0.30	0.37	0.25	-0.49*	0.23	-0.22	0.13	-0.45*	0.154	0.21	0.42*	0.26	1			
ABA	-0.59**	0.52**	-0.57**	-0.56**	0.23	-0.50*	0.04	-0.57**	-0.49*	-0.39*	-0.47*	-0.37	-0.36	0.40*	-0.14	0.23	0.41*	0.15	-0.09	-0.14	-0.51**	-0.41*	-0.21	1		
GA3	0.92**	-0.79**	0.91**	0.67**	-0.65**	0.70**	0.31	0.56**	0.77**	0.48*	0.70**	0.66**	0.54**	-0.55**	0.58**	-0.50*	-0.41*	-0.05	0.56**	0.62**	0.92**	0.41*	0.41*	-0.61**	1	
IAA	0.85**	-0.62**	0.78**	0.66**	-0.45*	0.77**	0.32	0.81**	0.47*	0.77**	0.89**	0.55**	0.30	-0.67**	0.61**	-0.39	-0.28	0.01	0.39	0.49*	0.82**	0.12	0.50*	-0.59**	0.72**	1
ZR	0.90**	-0.68**	0.89**	0.64**	-0.59**	0.73**	0.41*	0.65**	0.63**	0.58**	0.74**	0.69**	0.61**	-0.56**	0.59**	-0.68*	-0.34	-0.07	0.44*	0.70**	0.93**	0.32	0.50*	-0.48*	0.87**	0.81**

N=450; *$P < 0.05$, **$P < 0.01$. LI: leaf index; LA: leaf area; LT: leaf thickness; SLA: Specific Leaf Area; PT: Palisade tissue thickness; PSR: Ratio of palisade tissue to spongy tissue; MVBA: Main vein vascular bundle area; MXA: Main vein xylem area; XA/VBA: Main vein xylem/main vascular bundle area; VA: Vessel area; Pn: Photosynthetic rate; Tr: Transpiration rate; Gs: Stomatal conductance; C_i: Intercellular CO_2 concentration; RWC: Relative water content; LWP: Leaf water potential; WUE_i: Instantaneous water use efficiency;$\delta^{13}C$: Stable carbon isotope values; Pro: Proline; MDA: Malondialdehyde; SS: Soluble sugar; SP: Soluble protein; ABA: Abscisic acid; GA_3: Gibberellin; IAA: Indoleacetic acid; ZR: Zeatin Riboside.

Supplementary Table S3. Correlation analysis between tree height and structural and functional traits of heteromorphic leaves under drought stress.

R	H	LI	LA	LT	SLA	PT	PSR	MVBA	MXA	VBA/XA	VA	Pn	Tr	Ci	Gs	RWC	LWP	WUEi	δ¹³ C	Pro	MDA	SS	SP	ABA	GA3	IAA
LI	-0.73**	1																								
LA	0.67**	-0.36	1																							
LT	0.65*	-0.16	0.40*	1																						
SLA	0.34	0.55**	-0.09	-0.31	1																					
PT	0.87**	-0.66**	0.63**	0.27	0.24	1																				
PSR	0.82**	-0.57**	0.54**	0.07	0.24	0.71**	1																			
MVBA	0.76**	-0.75**	0.52**	0.29	-0.19	0.61**	0.61**	1																		
MXA	0.90**	-0.74**	0.50**	0.20	0.03	0.73**	0.81**	0.82**	1																	
XA /VBA	0.71*	-0.23	0.55*	0.31	0.23	0.24	0.12	0.25	0.36	1																
VA	0.95**	-0.69**	0.60**	0.19	0.28	0.73**	0.77**	0.77**	0.94**	0.51**	1															
Pn	0.73**	-0.38	0.58**	0.25	0.29	0.46*	0.63**	0.63**	0.74**	0.50*	0.82**	1														
Tr	0.65**	-0.42*	0.56**	0.28	0.04	0.47*	0.41*	0.66**	0.63**	0.05	0.62**	0.56**	1													
Ci	-0.70**	0.45*	-0.34	0.21	-0.59**	-0.60**	-0.59**	-0.29	-0.46*	-0.07	-0.57**	-0.31	-0.13	1												
Gs	0.45*	-0.15	0.63**	0.27	0.35	0.32	0.16	0.35	0.39	0.50**	0.50*	0.48*	0.54**	-0.08	1											
RWC	-0.04	-0.22	-0.07	0.01	-0.45*	-0.10	-0.10	0.32	0.14	-0.01	0.08	0.19	0.10	0.35	-0.13	1										
LWP	-0.39*	0.38	-0.28	-0.23	-0.09	-0.22	-0.3	-0.34	-0.37	-0.20	-0.39	-0.23	-0.30	0.27	-0.17	0.41*	1									
WUEi	0.07	-0.08	-0.01	-0.11	0.17	-0.09	-0.05	-0.07	0.09	0.39*	0.21	0.09	0.01	0.02	0.39	0.16	0.04	1								
δ¹³ C	-0.73*	0.63**	0.71*	0.18	0.23	-0.31	-0.18	-0.31	-0.29	-0.16	-0.23	0.06	0.12	0.42*	0.12	0.17	0.37	0.16	1							
Pro	0.71**	-0.32	0.60**	0.11	0.45*	0.51**	0.64**	0.54**	0.67**	0.32	0.73**	0.60**	0.48*	-0.48*	0.70**	-0.13	-0.34	0.13	-0.08	1						
MDA	0.43*	-0.23	0.41*	-0.22	-0.20	0.14	-0.14	0.35	0.10	0.10	0.15	0.12	0.18	-0.13	-0.01	0.23	0.07	0.18	-0.12	-0.18	1					
SS	0.01	-0.14	0.23	0.15	-0.13	0.07	-0.25	0.18	-0.01	0.01	-0.02	-0.17	0.29	0.18	0.59**	-0.04	-0.05	0.06	-0.14	0.32	-0.04	1				
SP	0.41*	-0.25	0.17	-0.06	0.25	0.17	0.18	0.40*	0.41*	0.31	0.52**	0.38	0.38	-0.29	0.52**	0.01	-0.31	0.26	-0.14	0.71**	0.14	0.38	1			
ABA	-0.86**	0.61**	-0.47*	0.04	-0.36	-0.69**	-0.73**	-0.53**	-0.72**	-0.06	-0.76**	-0.51**	-0.53**	0.80**	-0.13	0.16	0.36	-0.02	0.23	-0.49*	-0.17	0.23	-0.33	1		
GA3	0.81**	-0.55**	0.82**	0.45*	0.27	0.76**	0.65**	0.71**	0.75**	0.53**	0.79**	0.63**	0.53**	-0.39	0.68**	-0.04	-0.29	0.03	-0.21	0.74**	-0.07	0.28	0.28	-0.45*	1	
IAA	0.95**	-0.67**	0.72**	0.25	0.38	0.77**	0.72**	0.75**	0.85**	0.41*	0.93**	0.70**	0.70**	-0.61**	0.59**	-0.04	-0.45*	0.14	-0.19	0.78**	0.09	0.17	0.54**	-0.78**	0.84**	1
ZR	0.80**	-0.55**	0.76**	0.43*	0.23	0.74**	0.62**	0.71**	0.77**	0.53**	0.80**	0.62**	0.53**	-0.35	0.66**	-0.03	-0.30	0.04	-0.18	0.72**	-0.05	0.29	0.28	-0.44*	0.98**	0.84**

N=450; $^{*}P < 0.05$, $^{**}P < 0.01$. LI: leaf index; LA: leaf area; LT: leaf thickness; SLA: Specific Leaf Area; PT: Palisade tissue thickness; PSR: Ratio of palisade tissue to spongy tissue; MVBA: Main vein vascular bundle area; MXA: Main vein xylem area; XA/VBA: Main vein xylem/main vascular bundle area; VA: Vessel area; Pn: Photosynthetic rate; Tr: Transpiration rate; Gs: Stomatal conductance; C_i: Intercellular CO_2 concentration; RWC: Relative water content; LWP: Leaf water potential; WUE_i: Instantaneous water use efficiency;$\delta^{13}C$: Stable carbon isotope values; Pro: Proline; MDA: Malondialdehyde; SS: Soluble sugar; SP: Soluble protein; ABA: Abscisic acid; GA_3: Gibberellin; IAA: Indoleacetic acid; ZR: Zeatin Riboside.
